# Effect of Nebivolol and Olmesartan on 24-Hour Brachial and Aortic Blood Pressure in the Acute Stage of Ischemic Stroke

**DOI:** 10.1155/2019/9830295

**Published:** 2019-10-07

**Authors:** Eleni Georgianou, Panagiotis I. Georgianos, Konstantinos Petidis, Konstantinos Markakis, Ioanna Zografou, Asterios Karagiannis

**Affiliations:** ^1^2^nd^ Propedeutic Department of Medicine, Hippokration Hospital, Aristotle University of Thessaloniki, Thessaloniki, Greece; ^2^Section of Nephrology and Hypertension, 1^st^ Department of Medicine, AHEPA Hospital, Aristotle University of Thessaloniki, Thessaloniki, Greece

## Abstract

**Background:**

Elevated blood pressure (BP) in the acute phase of ischemic stroke is associated with heightened risk of early disability and death. However, whether BP-lowering in this setting is beneficial and the exact levels at which BP should be targeted remain unclear. This study aimed to evaluate the effect of nebivolol, olmesartan, and no-treatment on 24-hour BP in patients with hypertension during the acute poststroke period.

**Methods:**

In a single-blind fashion, 60 patients with acute ischemic stroke and clinic systolic BP (SBP) 160–220 mmHg were randomized to nebivolol (5 mg/day), olmesartan (20 mg/day), or no-treatment between Day 4 and Day 7 of stroke onset. BP-lowering efficacy was assessed through 24-hour BP monitoring using the Mobil-O-Graph device (IEM, Germany).

**Results:**

Between baseline and Day 7, significant reductions in 24-hour brachial SBP were noted with nebivolol and olmesartan, but not with no-treatment. Change from baseline (CFB) in 24-hour brachial SBP was not different between nebivolol and olmesartan groups (between-group difference: −3.4 mmHg; 95% confidence interval (CI): −11.2, 4.3), whereas nebivolol was superior to no-treatment in lowering 24-hour brachial SBP (between-group difference: −7.8 mmHg; 95% CI: −7.8 mmHg; 95% CI: −15.6, −0.1). Similarly, nebivolol and olmesartan equally lowered 24-hour aortic SBP (between-group difference: −1.9 mmHg; 95% CI: −10.1, 6.2). Nebivolol and olmesartan provoked similar reductions in 24-hour heart rate-adjusted augmentation index and pulse wave velocity.

**Conclusion:**

This study suggests that during the acute phase of ischemic stroke, nebivolol is equally effective with olmesartan in improving 24-hour aortic pressure and arterial stiffness indices. ClinicalTrials.gov identifier number: NCT03655964.

## 1. Introduction

Unlike the established benefit of blood pressure (BP) control in primary and secondary prevention of stroke, management of acute BP elevation in the early poststroke period is an area of controversy [1]. Acute hypertensive response is a very common manifestation, affecting up to 75% of patients presenting with acute stroke [2]. Observational studies reported a U-shaped relationship between BP and outcomes, suggesting that both low and high BPs in the acute phase of stroke are independently associated with excess risk for premature death and later death or dependency [3, 4]. Randomized trials and recent meta-analyses, however, showed that initiation of antihypertensive drug therapy in acute phase of ischemic stroke is not accompanied by improvement in short- and mid-term outcomes [1, 5, 6].

Assessment and management of hypertension in acute stroke is routinely relied on conventional clinic BP recordings. Furthermore, a vast majority of randomized trials in acute stroke also used automated clinic BP recordings when assessing BP-lowering response to antihypertensive therapy [7, 8]. It has to be noted, however, that the “gold-standard” method of 24-hour BP monitoring may offer several advantages in this setting, given that this technique remains unaffected by observer bias, measurement variability, and pressor effect of hospital environment [9]. Imaging studies showed that 24-hour BP correlates more closely than clinic BP with intracerebral microvascular damage and severity of brain edema [1]. A meta-analysis of observational studies showed that elevated 24-hour BP is closely associated with poor short-, mid-, and long-term functional outcome after an acute stroke [10], whereas clinic BP recorded on hospital admission is of no prognostic significance.

A recently introduced, validated, brachial cuff-based oscillometric device (Mobil-O-Graph, IEM, Stolberg, Germany) enables the noninvasive determination of brachial and aortic BP, wave reflection, and arterial stiffness indices under static and ambulatory conditions [11]. From a pathophysiological standpoint, compared with brachial BP, aortic BP can more accurately reflect the hemodynamic stress imposed on target organs [12]. Accumulated evidence suggests that aortic BP is a stronger predictor of future cardiovascular events [13, 14] and mortality and responds differentially from brachial BP to specific antihypertensive drug categories [15]. In particular, randomized trials showed that atenolol and some other *β*-blockers were equally effective with agents blocking the renin-angiotensin-aldosterone system (RAAS) in lowering brachial BP but less effective in improving central hemodynamic parameters [16, 17]. The latter may explain, at least partially, the inferiority of some *β*-blockers in cardiovascular risk reduction and overall hypertension management [15]. However, the natural course of 24-hour aortic BP, wave reflection, and arterial stiffness indices as well as their response to antihypertensive therapy in the acute stage of ischemic stroke was never previously investigated.

The aim of this study is to evaluate the effect of nebivolol, olmesartan, and no-treatment on 24-hour brachial and aortic BP, augmentation index (AIx), and pulse wave velocity (PWV) in patients presenting with raised BP during the acute stage of ischemic stroke.

## 2. Materials and Methods

### 2.1. Study Population

This is a single-blind, randomized, parallel-group, active-treatment controlled clinical study that was carried out in the 2^nd^ Propedeutic Department of Medicine, Hippokration University Hospital, Thessaloniki, Greece. We recruited 60 patients aged 18 years or older with transient ischemic attack or ischemic stroke, confirmed by computed tomography (CT) within 48 hours of symptom onset. Patients were eligible if their clinic BP at start of Day 3 of hospitalization was 160/100 mmHg or higher. Patients with clinic BP >220/120 mmHg or those with clinic BP >200/100 mmHg and evidence of acute target-organ damage (i.e., acute heart failure, myocardial infarction, unstable angina, aortic dissection, and acute renal failure) were excluded because current guidelines mandate immediate and aggressive BP-lowering in these individuals [18, 19]. Similarly, patients receiving intravenous thrombolytic therapy were excluded due to different guideline recommendations for BP-lowering in this setting [18, 19]. Additional prespecified exclusion criteria of the study were the following: (i) chronic atrial fibrillation or other cardiac arrhythmia; (ii) contraindication or definite clinical indication for treatment with a *β*-blocker; (iii) contraindication or definite clinical indication for treatment with an angiotensin receptor blocker; (iv) known allergic reaction to nebivolol or olmesartan; (v) body mass index (BMI) >40 kg/m^2^; and (vi) deep coma or dysphagia that disabled the oral administration of study drugs.

Protocol procedures were conducted in accordance with the Declaration of Helsinki and its latest amendments. Informed written consent was obtained from all study participants or their immediate family members before study enrollment. The study protocol was approved by the Ethics Committee of the School of Medicine, Aristotle University of Thessaloniki. The study is registered in ClinicalTrials.gov with the identifier number NCT03655964.

### 2.2. Randomization and Masking

Eligible patients were randomly assigned in a 1 : 1 : 1 ratio to single-blind therapy with nebivolol (5 mg/day), olmesartan (20 mg/day), or no antihypertensive treatment from Day 4 to Day 7 of ischemic stroke onset. Participants randomly assigned to no-treatment discontinued their home antihypertensive medications at hospital admission, if any, and did not receive BP-lowering medications throughout the study. Randomization was performed using a random permuted block design, and computer-generated random sequence was used for allocation concealment. Treating study physicians, nurses, and study investigators were not blinded to group assignment, whereas study participants were masked to treatment allocation.

### 2.3. Outcomes

Primary outcome of this study was the difference between nebivolol and olmesartan groups in the mean change from baseline (CFB) (Day 3) to study-end (Day 7) of 24-hour aortic systolic BP (SBP). The secondary outcome was defined as the mean difference between active treatment and no-treatment groups in CFB of 24-hour aortic SBP. Other secondary outcomes of this study included the mean difference between groups in CFB of 24-hour brachial BP, 24-hour heart rate-adjusted AIx (AIx(75)), and 24-hour PWV.

### 2.4. Data Collection and Patient Evaluation

Demographic characteristics, medical history, prestroke treatment for hypertension, and other comorbidities were collected at the time of patient enrollment. All data were captured on purpose-built data-collecting sheets. On admission, preexisting antihypertensive therapy, if any, was discontinued according to current guideline recommendations [18, 19]. Baseline evaluation of eligible patients who volunteered to participate in this study was performed at 07 : 00 h of Day 3 after stroke onset. Body weight and height were measured, and BMI was calculated as weight divided by height squared. Triplicate clinic BP measurements at the level of brachial artery were obtained with a Welch Allyn 7670-03 Mobile Aneroid Mercury sphygmomanometer and a cuff of appropriate size in the nonparetic arm to assess eligibility criteria [20]. Blood specimens were acquired for determination of routine hematological and biochemical parameters. After blood sampling, the Mobil-O-Graph device and a cuff of appropriate size were fitted in the nonparetic arm, and 24-hour BP monitoring was started.

After completing all these evaluations, participants were randomized to single-blind therapy with nebivolol, olmesartan, or no-treatment from Day 4 to Day 7 of stroke onset, following the above-described allocation concealment. All other routine aspects of the management of patients, including neuroimaging, acute treatment, and standard secondary prevention therapy, were managed at the discretion of treating physicians, according to currently available guidelines [18, 19]. Off-study administration of agents blocking the renin-angiotensin-aldosterone system (RAAS) and *β*-blockers was by protocol prohibited. Supervised administration of nebivolol and olmesartan during hospitalization by study nurses facilitated the adherence of study participants to the antihypertensive regimen. At Day 7 of hospitalization, all baseline evaluations were repeated. After study completion/hospital discharge, study participants were prescribed antihypertensive therapy, if necessary, according to guidelines [18, 19].

### 2.5. Brachial and Aortic BP Monitoring

Study participants underwent 24-hour brachial and aortic BP monitoring at baseline (Day 3) and study-end (Day 7), using the Mobil-O-Graph PWA device (IEM, Stolberg, Germany). The Mobil-O-Graph device is a brachial cuff-based oscillometric monitor, FDA- and CE-approved, and validated according to the British Hypertension Society and European Society of Hypertension protocols for brachial BP monitoring [21, 22]. The device was programmed to obtain 3 BP recordings per hour during day-time (07 : 00–22 : 59) and 2 BP recordings per hour during night-time (23 : 00–06 : 59). The methodology incorporated by this device was previously described [23]. In brief, after the conventional brachial BP recording, the brachial cuff reinflates at the level of diastolic BP (DBP), acquiring the brachial pressure waveforms for approximately 10 seconds. Afterwards, an aortic pulse waveform is generated via a generalized transfer function algorithm (ARCSolver algorithm) [23]. Calibration of the aortic pulse waveform was performed using brachial SBP/DBP, as previously described elsewhere [23]. Pulse pressure (PP) was calculated as SBP minus DBP. Mean BP (MBP), the underlying principle of the oscillometric method, defined as the lowest cuff pressure at which the oscillations are maximal, was also measured. Wave separation analysis was also performed by decomposing the aortic pulse waveform into forward-traveling (incident) and backward-traveling (reflected) pulse waves with a triangular aortic flow waveform. ARCSolver algorithm estimates AIx(75) and PWV utilizing parameters from pulse wave analysis and wave separation analysis [23]. The precision and accuracy of Mobil-O-Graph-derived central hemodynamic indices has been validated under static conditions against standardized noninvasive tonometric measurements (Sphygmocor, ArtCor, Sydney, Australia) [24, 25] as well as against “gold-standard” intra-aortic measurements with consistently accurate results [26]. This device has been shown to provide highly reproducible estimations of central hemodynamic indices under ambulatory conditions, as well [27].

### 2.6. Statistical Analysis

Statistical analysis was performed with Statistical Package for Social Sciences 23 (SPSS Inc., Chicago, Illinois, USA). Continuous variables are expressed as mean ± standard deviation (mean ± SD) and categorical variables as absolute frequencies and percentages (*n*, %). The normality of distribution of variables was assessed using the Kolmogorov–Smirnov test. Differences among treatment groups in baseline characteristics were evaluated with one-way analysis of variance (ANOVA) for continuous variables and chi-squared (*χ*^2^) test for categorical variables. For comparisons between baseline and study-end in each treatment group, paired Student's *t*-tests or Wilcoxon's signed rank tests were applied, according to the normality of distribution. Between-group differences in CFB of continuous variables were evaluated with univariate analysis of covariance (ANCOVA). In ANCOVA models, treatment group was inserted as a fixed-effect factor, and baseline value of each variable was inserted as a covariate. Between-group differences are expressed as mean values with 95% confidence interval (CI). Probability values of *P* < 0.05 (two-tailed) were considered statistically significant.

Sample size calculation was carried out with nQuery advisor version 5.0. software (Statistical Solutions, Boston, MA). This pilot study had above 80% statistical power to detect a difference of 4.0 mmHg in CFB of 24-hour aortic SBP between the nebivolol and olmesartan groups with *a* = 0.05 and assuming an SD for this difference of 8.0 mmHg.

## 3. Results

### 3.1. Patient Enrollment and Baseline Characteristics

**Figure 1:**
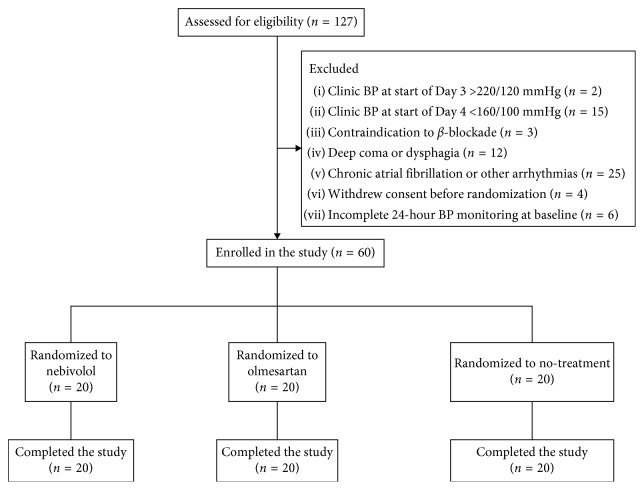
Flow diagram of patient enrollment.

The trial flow diagram of patient enrollment is depicted in Figure 1. Among 127 hospitalized patients screened, 67 patients were not eligible in the study for the following reasons: (i) 25 patients due to chronic atrial fibrillation or other cardiac arrhythmia; (ii) 18 patients due to clinic BP at start of Day 3 that was out of the prespecified range of 160–220/100–120 mmHg; (iii) 12 patients due to deep coma or dysphagia that disabled the oral administration of study drugs; (iv) 6 patients due to incomplete 24-hour BP monitoring at baseline; (v) 4 patients withdrew consent before randomization; and (vi) 3 patients had contraindication to receive *β*-blockade. A total of 60 patients with hypertension in the acute stage of ischemic stroke were finally randomized and completed the study.

The baseline characteristics of study participants are presented in Table 1. Study population consisted of 22 men and 38 women with a mean age of 79.3 ± 8.2 years and a mean BMI of 26.5 ± 3.5 kg/m^2^. Preexisting hypertension and history of previous stroke had 77.3% and 23.3% of study participants, respectively. The overall baseline 24-hour brachial SBP was 153.1 ± 16.9 mmHg and did not significantly differ among treatment groups. Although history of preexisting hypertension, stroke, and coronary artery disease was balanced among groups, patients randomized to no-treatment tended to be more commonly males and had more commonly history of diabetes. Routine laboratory parameters and serum lipid profile were not different among groups (Table 1).

### 3.2. Treatment Effect on 24-Hour Brachial BP

As shown in Table 2, significant reductions in 24-hour brachial SBP between baseline and Day 7 were noted in nebivolol (153.4 ± 19.1 vs. 141.1 ± 15.6 mmHg, *P* < 0.001) and olmesartan group (151.7 ± 14.0 vs. 143.4 ± 16.9 mmHg, *P*=0.032). In the no-treatment group, 24-hour brachial SBP fell from 154.3 ± 18.2 to 149.6 ± 19.2 mmHg, but this drop of 4.7 mmHg did not reach statistical significance (*P*=0.08). As shown in Table 3, CFB of 24-hour brachial SBP did not differ between nebivolol and olmesartan groups (between-group difference: −3.4 mmHg; 95% CI: −11.2, 4.3; *P*=0.37) as well as between olmesartan and no-treatment groups (between-group difference: −4.3 mmHg; 95% CI: −12.1, 3.4; *P*=0.27). By contrast, nebivolol was superior to no-treatment in lowering 24-hour brachial SBP (between-group difference: −7.8 mmHg; 95% CI: −15.6, −0.1; *P*=0.049). Between baseline and Day 7, significant reductions in 24-hour brachial DBP and PP were observed in nebivolol and olmesartan groups, but not in no-treatment group. CFB of 24-hour brachial DBP and PP were not different among groups. As expected, a significant reduction in 24-hour heart rate between Day 3 to Day 7 was evident in the nebivolol group (73.8 ± 12.4 vs. 70.3 ± 13.7 bpm, *P*=0.030), whereas heart rate remained unchanged with olmesartan and no-treatment.

### 3.3. Treatment Effect on Central Hemodynamic Parameters

Similarly to brachial pressures, a significant reduction in 24-hour aortic SBP was observed between baseline and Day 7 in nebivolol (137.9 ± 18.7 vs. 126.1 ± 14.2 mmHg, *P* < 0.001) and olmesartan groups (140.9 ± 13.5 vs. 130.1 ± 15.5 mmHg, *P*=0.006), whereas the drop of 24-hour aortic SBP from 140.3 ± 16.7 to 135.1 ± 19.9 mmHg with no-treatment was not significant (Table 2). CFB of 24-hour aortic SBP did not differ between nebivolol and olmesartan groups (between-group difference: −1.9 mmHg; 95% CI: −10.2, 6.2; *P*=0.63) as well as between the olmesartan and no-treatment groups (between-group difference: −5.4 mmHg; 95% CI: −13.6, 2.7, *P*=0.19). However, compared with no-treatment, nebivolol induced a significantly higher reduction in 24-hour aortic SBP (between-group difference: −7.3 mmHg; 95% CI: −12.5, −0.1, *P*=0.048). Between baseline and Day 7, significant reductions in 24-hour aortic DBP and PP were noted in the active-treatment groups, but not with no-treatment. These reductions were numerically higher in the nebivolol group but did not significantly differ from corresponding changes in olmesartan and no-treatment groups. Nebivolol and olmesartan had also no effect on the amplification of PP between the aorta and brachial artery (Tables 2 and 3).

With respect to wave reflections, significant reductions in 24-hour AIx(75) between baseline and Day 7 were observed in both nebivolol (34.0 ± 6.8 vs. 31.7 ± 8.7%, *P*=0.047) and olmesartan groups (34.7 ± 5.5 vs. 31.2 ± 7.4%, *P*=0.038), whereas 24-hour AIx(75) remained constant throughout the study in the no-treatment group. Similarly, 24-hour PWV was significantly reduced by 0.8 m/sec in response to nebivolol and olmesatan therapy, but remained unchanged with no-treatment (Table 2).

### 3.4. Adverse Events

With the exception of one episode of orthostatic hypotension in the no-treatment group and one episode of fall without fracture in the nebivolol group, no other serious adverse events were recorded during follow-up. No study participant withdrew consent after randomization due to side effects.

## 4. Discussion

This single-blind, randomized study aimed to evaluate the effect of nebivolol, olmesartan, and no-treatment on 24-hour brachial and aortic BP applying for first time the newly introduced Mobil-O-Graph monitor in patients presenting with hypertension after an acute ischemic stroke. The main findings of this study were the following: (i) between Day 3 and Day 7 of stroke onset, 24-hour brachial BP fell by 12.2/4.8, 8.3/2.6, and 4.7/1.9 mmHg in the nebivolol, olmesartan, and no-treatment groups, respectively, suggesting a potentially more potent BP-lowering effect of nebivolol in the acute poststroke period; (ii) despite the nebivolol-induced reduction in 24-hour heart rate, nebivolol and olmesartan induced comparable reductions in 24-hour aortic SBP, AIx(75), and PWV, suggesting a favorable effect of nebivolol on central hemodynamics that differentiates this agent from other nonvasodilating *β*-blockers.

Earlier meta-analyses of randomized trials quantifying the comparative effectiveness of monotherapy with different antihypertensive drug categories on brachial versus aortic BP showed that *β*-blockers are inferior to RAAS blockers and other antihypertensives in lowering aortic SBP and AIx [28, 29]. The inferiority of *β*-blockers to improve central hemodynamics is mainly attributed to the treatment-induced reduction in heart rate that affects the timing of synchronization between incident and backward-traveling pulse waves in the ascending aorta [17]. It has to be noted, however, that less intensive lowering of aortic pressures should not be considered a unique class effect of all *β*-blockers. Pilot randomized trials conducted in the general hypertensive population support the notion that the third-generation, vasodilating *β*-blocker nebivolol exerts a beneficial action on reflecting properties of microcirculation that is translated into equally effective lowering of brachial and aortic pressures. For example, Kampus et al. [30] randomized 80 drug-naive hypertensives to double-blind therapy with nebivolol (5 mg/day) or metoprolol (50–100 mg/day). Although both agents equally lowered brachial SBP and heart rate during the 12-month-long follow-up, a significant reduction of 12.4 mmHg in aortic SBP was noted only in nebivolol-treated participants [30]. Another trial randomized 40 drug-naïve hypertensives to nebivolol (5 mg/day) or atenolol (50 mg/day) for 4 weeks [31]. Once again, both *β*-blockers induced equal reductions in brachial BP, but nebivolol was superior to atenolol in lowering aortic PP and AIx [31]. The present study expands these observations, showing an acute beneficial action of nebivolol on AIx(75) and aortic SBP using the method of 24-hour pulse wave analysis. The acceptable accuracy and reproducibility of this method offers several advantages over standardized measurement of vascular biomarkers in static (office) conditions [27, 32]. Estimation of office AIx and PWV is largely operator-dependent, and these indices cannot accurately reflect the circadian fluctuation of vascular biomarkers during the 24-hour period [33]. Compared with office PWV, ambulatory PWV is shown to be more closely associated with indices of hypertension-related target-organ damage [34]. Longitudinal studies have provided evidence supporting that ambulatory PWV is a strong predictor of all-cause and cardiovascular mortality with a predictive value that extends above and beyond office PWV [35].

Nebivolol-induced reduction in 24-hour aortic SBP and AIx(75) in the present study may be—at least partially—explained by the pharmacological properties and the unique mechanism of action of this agent. In sharp contrast to older cardioselective *β*-blockers, nebivolol displays vasodilatation properties mediated through the L-arginine-nitric oxide-dependent pathway [36, 37]. Nebivolol improves endothelium-dependent vasodilatation by enhancing nitric oxide (NO) production through a stimulatory effect on constitutive NO-synthase activity as well as by downregulating oxidative inactivation of NO [36, 37]. Increased NO availability may modify the vascular tone of small resistance arteries, explaining the nebivolol-inducible reduction in AIx(75). Moreover, the vasodilatation properties of nebivolol may lie behind the less potent chronotropic action of this agent. Compared with other *β*-blockers, nebivolol was shown to exert a milder heart rate-lowering effect, providing an alternative explanation for the preferential improvement in wave reflections with this *β*-blocker [36, 37]. All these actions of nebivolol may be particularly applicable to patients with acute hypertensive response in the early poststroke period, given that this condition is pathophysiologically characterized by excessive vasoconstriction mediated through autonomic dysfunction, sympathetic overactivity, and raised levels of circulatory catecholamines and brain natriuretic peptides [38].

Whether BP-lowering in the acute stage of ischemic stroke is translated into benefit on functional outcome and mortality risk is an issue surrounded by controversy. In a 2014 Cochrane meta-analysis of 26 randomized trials (incorporating data from 17,011 participants), active therapy was superior to placebo in lowering BP levels within 24 hours after randomization [5]. BP-lowering responses were similar regardless of antihypertensive drug class. However, active therapy was not superior to placebo in lowering the risk of premature death or dependency after an acute stroke [5]. An earlier metaregression analysis of 37 randomized trials (involving 9,008 participants) uncovered the presence of a U-shaped or J-shaped association between treatment-induced change in BP levels and the risk for subsequent death or dependency [6]. A post hoc analysis of 2,029 patients participating in the Scandinavian Candesartan Acute Stroke Trial (SCAST) confirmed that large decreases as well as no change/increase in SBP during the acute stage of stroke were both associated with higher risk for early adverse events and poor neurological outcome [39]. On this basis, currently available guidelines mandate the delayed administration of antihypertensive therapy in the hyperacute phase of ischemic stroke [18, 19], but the exact levels at which BP should be targeted in the following days of stroke onset still remain elusive.

The results of this pilot randomized study should be interpreted within the context of the strengths and limitations of its design. Taking into consideration the excessive short-term and day-to-day variability of BP in the acute stage of stroke and the potential influence of hospital environment on BP recordings [40, 41], we used the “gold-standard” method of 24-hour BP monitoring as the most objective approach to quantify on-treatment alterations in BP levels [9]. Furthermore, we applied the novel and validated Mobil-O-Graph device that enabled the continuous monitoring of central hemodynamic parameters under static and ambulatory conditions—an advantage over conventional brachial BP monitoring. This study also has some limitations that need to be acknowledged. Study investigators and physicians were not blinded to treatment allocation, and placebo was not administered to patients randomized to no-treatment. Despite the fact that the technique of 24-hour BP monitoring minimizes the placebo effect [42], the results of the present study warrant confirmation by future double-blind and placebo-controlled trials. In addition, the design of this study did not prespecify the prolonged follow-up of study participants after the end of the intervention or hospital discharge. Accordingly, we did not capture long-term alterations in neurological status, and we did not record the incidence of other clinically relevant endpoints (i.e., mortal events, stroke recurrence, and other cardiovascular events). Third, the sample size of this study did not provide adequate statistical power in order to detect small between-group differences in the primary outcome. The difference of 1.9 mmHg in the CFB of 24-hour aortic SBP between the nebivolol and olmesartan groups was not statistically significant in our analysis, but this difference may still be of clinical relevance. Future studies with larger samples sizes and longer observational periods are necessary in order to evaluate the prognostic significance of on-treatment change in 24-hour aortic BP and AIx among patients with acute ischemic stroke. Finally, treatment effects of nebivolol and olmesartan on indices of short-term BP variability were not evaluated in this paper but will be the subject of future analyses of our data.

## 5. Conclusion

In conclusion, this study suggests that the third-generation, vasodilating *β*-blocker nebivolol is equally effective with olmesartan in lowering 24-hour aortic BP, AIx(75), and PWV. The vasodilating properties of nebivolol and its favorable impact on central hemodynamic indices suggest a potential role of this agent in the management of hypertension in the acute stage of ischemic stroke that warrants further investigation in larger and properly designed trials.

## Figures and Tables

**Table 1:** Baseline, demographic, clinical, and laboratory characteristics of study participants.

Parameter	Overall (*n*=60)	Nebivolol (5 mg/day) (*n*=20)	Olmesartan (20 mg/day) (*n*=20)	No-treatment (*n*=20)	*P* value
Age (years)	79.3 ± 8.2	80.7 ± 7.3	79.5 ± 9.5	77.7 ± 7.8	0.51
Male gender (*n*, %)	22 (36.7)	5 (25.0)	6 (30.0)	11 (55.0)	0.11
Body weight (kg)	73.3 ± 9.6	75.2 ± 11.3	70.5 ± 7.5	74.2 ± 9.7	0.27
Height (m)	1.66 ± 0.1	1.65 ± 0.1	1.66 ± 0.1	1.68 ± 0.1	0.46
BMI (kg/m^2^)	26.5 ± 3.5	27.5 ± 3.8	25.5 ± 3.6	26.4 ± 3.1	0.21
History of DM (*n*, %)	24 (40.0)	7 (35.0)	5 (25.0)	12 (60.0)	0.07
History of hypertension (*n*, %)	44 (73.3)	13 (65.0)	16 (80.0)	15 (75.0)	0.55
History of CAD (*n*, %)	11 (18.3)	5 (25.0)	3 (15.0)	3 (15.0)	0.36
History of previous stroke (*n*, %)	14 (23.3)	6 (30.0)	2 (10.0)	6 (30.0)	0.19
24-h brachial SBP (mmHg)	153.1 ± 16.9	153.4 ± 19.1	151.7 ± 14.0	154.3 ± 18.2	0.89
24-h brachial DBP (mmHg)	84.4 ± 9.9	83.1 ± 9.2	84.7 ± 7.5	85.4 ± 12.8	0.76
Serum urea (mg/dl)	45.8 ± 15.8	46.3 ± 17.7	45.5 ± 13.3	45.6 ± 16.9	>0.90
Serum creatinine (mg/dl)	1.1 ± 0.2	1.1 ± 0.3	1.0 ± 0.2	1.1 ± 0.2	0.84
Uric acid (mg/dl)	4.9 ± 1.3	4.9 ± 1.1	5.0 ± 1.7	4.7 ± 1.3	0.72
Total cholesterol (mg/dl)	173.4 ± 44.5	160.8 ± 41.1	190.6 ± 39.3	168.9 ± 48.8	0.09
HDL cholesterol (mg/dl)	46.7 ± 11.8	44.4 ± 9.4	50.5 ± 13.5	45.4 ± 11.8	0.21
LDL cholesterol (mg/dl)	113.9 ± 39.3	104.5 ± 33.1	118.1 ± 35.2	119,1 ± 48.2	0.43
Triglycerides (mg/dl)	121.8 ± 44.0	112.2 ± 43.7	121.6 ± 46.3	131.6 ± 42.0	0.38

BMI = body mass index; CAD = coronary artery disease; DM = diabetes mellitus; DBP = diastolic blood pressure; HDL = high-density lipoprotein; LDL = low-density lipoprotein; SBP = systolic blood pressure; data are presented as mean ± SD or absolutes frequencies and percentages.

**Table 2:** Comparisons of peripheral and central hemodynamic parameters between baseline and study-end by treatment group.

Parameter	Nebivolol 5 mg/day (*n*=20)			Olmesartan 20 mg/day (*n*=20)			No-treatment (*n*=20)		
	Baseline	Day 7	*P* value	Baseline	Day 7	*P* value	Baseline	Day 7	*P* value
24-h brachial SBP (mmHg)	153.4 ± 19.1	141.1 ± 15.6	**<0.001**	151.7 ± 14.0	143.4 ± 16.9	**0.032**	154.3 ± 18.2	149.6 ± 19.2	0.08
24-h brachial DBP (mmHg)	83.1 ± 9.2	78.5 ± 8.8	**0.001**	84.7 ± 7.5	82.0 ± 8.5	**0.011**	85.4 ± 12.9	83.3 ± 12.0	0.13
24-h brachial PP (mmHg)	70.3 ± 14.5	62.6 ± 12.1	**0.008**	67.0 ± 10.2	61.4 ± 11.3	**0.044**	68.9 ± 11.9	66.3 ± 12.3	0.27
24-h heart rate (bpm)	73.8 ± 12.4	70.3 ± 13.7	**0.030**	71.9 ± 10.2	71.7 ± 10.9	>0.90	72.7 ± 14.3	74.2 ± 17.4	0.52
24-h MBP (mmHg)	114.1 ± 13.1	105.7 ± 9.8	**<0.001**	114.4 ± 9.7	109.0 ± 12.8	**0.024**	117.0 ± 14.4	114.7 ± 15.0	0.26
24-h aortic SBP (mmHg)	137.9 ± 18.7	126.1 ± 14.2	**<0.001**	140.9 ± 13.5	130.1 ± 15.5	**0.006**	140.3 ± 16.7	135.1 ± 19.9	0.10
24-h aortic DBP (mmHg)	85.6 ± 9.5	80.9 ± 9.7	**0.001**	86.9 ± 8.2	83.5 ± 9.5	**0.042**	87.1 ± 12.8	86.1 ± 13.9	0.55
24-h aortic PP (mmHg)	52.2 ± 13.9	45.2 ± 11.5	**0.001**	53.9 ± 9.3	46.5 ± 9.2	**0.018**	53.2 ± 9.9	49.0 ± 10.3	0.12
24-h aortic-to-brachial PP amplification (mmHg)	18.1 ± 7.7	17.4 ± 7.2	0.45	13.1 ± 7.1	14.9 ± 7.6	0.42	15.7 ± 4.9	17.3 ± 6.8	0.18
24-h AIx(75) (%)	34.0 ± 6.8	31.7 ± 8.7	**0.047**	34.7 ± 5.5	31.2 ± 7.4	**0.038**	34.1 ± 7.5	32.9 ± 10.0	0.40
24-h PWV (m/sec)	12.8 ± 1.8	12.0 ± 1.9	**0.014**	13.4 ± 2.3	12.6 ± 2.8	**0.015**	12.7 ± 2.0	12.2 ± 2.4	0.10

AIx(75) = heart rate-adjusted augmentation index; DBP = diastolic blood pressure; MBP = mean blood pressure; SBP = systolic blood pressure; PP = pulse pressure; PWV = pulse wave velocity; data are presented as mean ± SD.

**Table 3:** Between-group differences in change from baseline to study-end of peripheral and central hemodynamic parameters.

Parameter	Nebivolol vs. olmesartan		Nebivolol vs. no-treatment		Olmesartan vs. no-treatment	
	MD (95% CI)	*P* value	MD (95% CI)	*P* value	MD (95% CI)	*P* value
24-h brachial SBP (mmHg)	−3.4 (−11.2, 4.3)	0.37	−7.8 (−15.6, −0.1)	**0.049**	−4.3 (−12.1, 3.4)	0.27
24-h brachial DBP (mmHg)	−2.2 (−6.0, 1.6)	0.25	−2.9 (−6.7, 0.9)	0.14	−0.7 (−4.5, 3.1)	0.73
24-h brachial PP (mmHg)	−0.6 (−6.9, 5.6)	0.83	−4.6 (−10.8, 1.6)	0.14	−3.9 (−10.1, 2.3)	0.21
24-h heart rate (bpm)	−2.8 (−9.7, 4.1)	0.42	−4.8 (−11.6, −0.1)	**0.045**	−1.9 (−8.8, 4.9)	0.57
24-h MBP (mmHg)	−3.2 (−9.5, 3.1)	0.31	−7.1 (−13.4, −0.8)	**0.027**	−3.9 (−10.2, 2.3)	0.21
24-h aortic SBP (mmHg)	−1.9 (−10.1, 6.2)	0.63	−7.3 (−12.5, −0.1)	**0.048**	−5.4 (−13.6, 2.7)	0.19
24-h aortic DBP (mmHg)	−1.6 (−6.4, 3.2)	0.50	−4.0 (−8.8, 0.8)	0.10	−2.4 (−7.2, 2.4)	0.32
24-h aortic PP (mmHg)	−0.6 (−6.5, 5.4)	0.85	−3.3 (−9.3, 2.6)	0.26	−2.8 (−8.8, 3.2)	0.35
24-h aortic-to-brachial PP amplification (mmHg)	−0.2 (−4.4, 3.9)	0.92	−1.3 (−5.3, 2.7)	0.52	−1.1 (−5.1, 2.9)	0.59
24-h AIx(75) (%)	1.2 (−2.8, 5.2)	0.54	−1.1 (−5.1, 2.8)	0.57	−2.4 (−6.3, 1.6)	0.24
24-h PWV (m/sec)	0.1 (−0.9, 0.9)	>0.90	−0.3 (−1.2, 0.7)	0.57	−0.3 (−1.2, 0.7)	0.57

AIx(75) = heart rate-adjusted augmentation index; CI = confidence interval; DBP = diastolic blood pressure; MBP = mean blood pressure; MD = mean difference; PP = pulse pressure; PWV = pulse wave velocity; SBP = systolic blood pressure; data are derived from univariate ANCOVA and are presented as mean between-group differences with corresponding 95% CI.

## Data Availability

The data used to support the findings of this study are available from the corresponding author upon request.

## References

[1] Gasecki D., Coca A., Cunha P. (2018). Blood pressure in acute ischemic stroke: challenges in trial interpretation and clinical management: position of the ESH working group on hypertension and the brain. *Journal of Hypertension*.

[2] Qureshi A. I., Ezzeddine M. A., Nasar A. (2007). Prevalence of elevated blood pressure in 563704 adult patients with stroke presenting to the ED in the United States. *The American Journal of Emergency Medicine*.

[3] Sare G. M., Ali M., Shuaib A., Bath P. M. W. (2009). Relationship between hyperacute blood pressure and outcome after ischemic stroke: data from the VISTA collaboration. *Stroke*.

[4] Tsivgoulis G., Spengos K., Vemmos K. N. (2004). Blood pressure in acute stroke and its prognostic value. *Stroke*.

[5] Bath P. M., Krishnan K. (2014). Interventions for deliberately altering blood pressure in acute stroke. *Cochrane Database of Systematic Reviews*.

[6] Geeganage C. M., Bath P. M. W. (2009). Relationship between therapeutic changes in blood pressure and outcomes in acute stroke: a metaregression. *Hypertension*.

[7] He J., Zhang Y., Xu T. (2014). Effects of immediate blood pressure reduction on death and major disability in patients with acute ischemic stroke: the CATIS randomized clinical trial. *JAMA*.

[8] Sandset E. C., Bath P. M., Boysen G. (2011). The angiotensin-receptor blocker candesartan for treatment of acute stroke (SCAST): a randomised, placebo-controlled, double-blind trial. *The Lancet*.

[9] Parati G., Stergiou G., O’Brien E (2014). European Society of Hypertension practice guidelines for ambulatory blood pressure monitoring. *Journal of Hypertension*.

[10] Kakaletsis N., Ntaios G., Milionis H (2015). Prognostic value of 24-h ABPM in acute ischemic stroke for short-, medium-, and long-term outcome: a systematic review and meta-analysis. *International Journal of Stroke*.

[11] Protogerou A. D., Smulyan H., Safar M. E. (2011). Closer to noninvasive out-of-office aortic blood pressure assessment: a time to think and act. *Hypertension*.

[12] Kollias A., Lagou S., Zeniodi M. E., Boubouchairopoulou N., Stergiou G. S. (2016). Association of central versus brachial blood pressure with target-organ damage: systematic review and meta-analysis. *Hypertension*.

[13] Pini R., Cavallini M. C., Palmieri V. (2008). Central but not brachial blood pressure predicts cardiovascular events in an unselected geriatric population: the ICARe Dicomano Study. *Journal of the American College of Cardiology*.

[14] Vlachopoulos C., Aznaouridis K., O’Rourke M. F., Safar M. E., Baou K., Stefanadis C. (2010). Prediction of cardiovascular events and all-cause mortality with central haemodynamics: a systematic review and meta-analysis. *European Heart Journal*.

[15] McEniery C. M., Cockcroft J. R., Roman M. J., Franklin S. S., Wilkinson I. B. (2014). Central blood pressure: current evidence and clinical importance. *European Heart Journal*.

[16] Asmar R. G., London G. M., O’Rourke M. E., Safar M. E. (2001). Improvement in blood pressure, arterial stiffness and wave reflections with a very-low-dose perindopril/indapamide combination in hypertensive patient: a comparison with atenolol. *Hypertension*.

[17] Pannier B. M., Guerin A. P., Marchais S. J., London G. M. (2001). Different aortic reflection wave responses following long-term angiotensin-converting enzyme inhibition and beta-blocker in essential hypertension. *Clinical and Experimental Pharmacology and Physiology*.

[18] Jauch E. C., Saver J. L., Adams H. P. (2013). Guidelines for the early management of patients with acute ischemic stroke: a guideline for healthcare professionals from the American Heart Association/American Stroke Association. *Stroke*.

[19] Powers W. J., Rabinstein A. A., Ackerson T. (2018). Guidelines for the early management of patients with acute ischemic stroke: a guideline for healthcare professionals from the American heart association/American stroke association. *Stroke*.

[20] Mancia G., Fagard R., Narkiewicz K. (2013). ESH/ESC guidelines for the management of arterial hypertension: the task force for the management of arterial hypertension of the European Society of Hypertension (ESH) and of the European Society of Cardiology (ESC). *Journal of Hypertension*.

[21] Franssen P. M., Imholz B. P. (2010). Evaluation of the Mobil-O-Graph new generation ABPM device using the ESH criteria. *Blood Pressure Monitoring*.

[22] Wei W., Tölle M., Zidek W., van der Giet M. (2010). Validation of the mobil-O-Graph: 24 h-blood pressure measurement device. *Blood Pressure Monitoring*.

[23] Papaioannou T. G., Protogerou A. D., Stamatelopoulos K. S., Vavuranakis M., Stefanadis C. (2009). Non-invasive methods and techniques for central blood pressure estimation: procedures, validation, reproducibility and limitations. *Current Pharmaceutical Design*.

[24] Papaioannou T. G., Protogerou A., Stefanadis C. (2012). Comparison between Mobil-O-Graph and the SphygmoCor device for central systolic blood pressure estimation: consensus is required for “validation protocols”. *Blood Pressure Monitoring*.

[25] Sarafidis P. A., Georgianos P. I., Karpetas A (2014). Evaluation of a novel brachial cuff-based oscillometric method for estimating central systolic pressure in hemodialysis patients. *American Journal of Nephrology*.

[26] Weber T., Wassertheurer S., Rammer M (2011). Validation of a brachial cuff-based method for estimating central systolic blood pressure. *Hypertension*.

[27] Protogerou A. D., Argyris A., Nasothimiou E. (2012). Feasibility and reproducibility of noninvasive 24-h ambulatory aortic blood pressure monitoring with a brachial cuff-based oscillometric device. *American Journal of Hypertension*.

[28] Manisty C. H., Hughes A. D. (2013). Meta-analysis of the comparative effects of different classes of antihypertensive agents on brachial and central systolic blood pressure, and augmentation index. *British Journal of Clinical Pharmacology*.

[29] McGaughey T. J., Fletcher E. A., Shah S. A. (2016). Impact of antihypertensive agents on central systolic blood pressure and augmentation index: a meta-analysis. *American Journal of Hypertension*.

[30] Kampus P., Serg M., Kals J (2011). Differential effects of nebivolol and metoprolol on central aortic pressure and left ventricular wall thickness. *Hypertension*.

[31] Mahmud A., Feely J. (2008). Beta-blockers reduce aortic stiffness in hypertension but nebivolol, not atenolol, reduces wave reflection. *American Journal of Hypertension*.

[32] Omboni S., Posokhov I. N., Kotovskaya Y. V., Protogerou A. D., Blacher J. (2016). Twenty-four-hour ambulatory pulse wave analysis in hypertension management: current evidence and perspectives. *Current Hypertension Reports*.

[33] Argyris A. A., Nasothimiou E., Aissopou E. (2018). Mechanisms of pulse pressure amplification dipping pattern during sleep time: the SAFAR study. *Journal of the American Society of Hypertension*.

[34] Aissopou E. K., Argyris A. A., Nasothimiou E. G. (2016). Ambulatory aortic stiffness is associated with narrow retinal arteriolar caliber in hypertensives: the SAFAR study. *American Journal of Hypertension*.

[35] Matschkal J., Mayer C. C., Sarafidis P. A (2019). Comparison of 24-hour and office pulse wave velocity for prediction of mortality in hemodialysis patients. *American Journal of Nephrology*.

[36] Agabiti-Rosei E., Porteri E., Rizzoni D. (2009). Arterial stiffness, hypertension, and rational use of nebivolol. *Vascular Health and Risk Management*.

[37] Borghi C., Acelajado M. C., Gupta Y., Jain S. (2017). Role of nebivolol in the control and management of central aortic blood pressure in hypertensive patients. *Journal of Human Hypertension*.

[38] Qureshi A. I. (2008). Acute hypertensive response in patients with stroke: pathophysiology and management. *Circulation*.

[39] Sandset E. C., Murray G. D., Bath P. M., Kjeldsen S. E., Berge E. (2012). Relation between change in blood pressure in acute stroke and risk of early adverse events and poor outcome. *Stroke*.

[40] Fukuda K., Kai H., Kamouchi M (2015). Day-by-day blood pressure variability and functional outcome after acute ischemic stroke: fukuoka stroke registry. *Stroke*.

[41] Manning L. S., Rothwell P. M., Potter J. F., Robinson T. G. (2015). Prognostic significance of short-term blood pressure variability in acute stroke: systematic review. *Stroke*.

[42] Mancia G., Omboni S., Parati G., Ravogli A., Villani A., Zanchetti A. (1995). Lack of placebo effect on ambulatory blood pressure. *American Journal of Hypertension*.

